# Assessing Syndromic Surveillance of Cardiovascular Outcomes from Emergency Department Chief Complaint Data in New York City

**DOI:** 10.1371/journal.pone.0014677

**Published:** 2011-02-14

**Authors:** Robert W. Mathes, Kazuhiko Ito, Thomas Matte

**Affiliations:** 1 Bureau of Environmental Surveillance and Policy, New York City Department of Health and Mental Hygiene, New York, New York, United States of America; 2 Environmental Medicine, New York University School of Medicine, Tuxedo, New York, United States of America; 3 Hunter College, City University of New York School of Public Health, New York, New York, United States of America; Universidad Peruana Cayetano Heredia, Peru

## Abstract

**Background:**

Prospective syndromic surveillance of emergency department visits has been used for near-real time tracking of communicable diseases to detect outbreaks or other unexpected disease clusters. The utility of syndromic surveillance for tracking cardiovascular events, which may be influenced by environmental factors and influenza, has not been evaluated. We developed and evaluated a method for tracking cardiovascular events using emergency department free-text chief complaints.

**Methodology/Principal Findings:**

There were three phases to our analysis. First we applied text processing algorithms based on sensitivity, specificity, and positive predictive value to chief complaint data reported by 11 New York City emergency departments for which ICD-9 discharge diagnosis codes were available. Second, the same algorithms were applied to data reported by a larger sample of 50 New York City emergency departments for which discharge diagnosis was unavailable. From this more complete data, we evaluated the consistency of temporal variation of cardiovascular syndromic events and hospitalizations from 76 New York City hospitals. Finally, we examined associations between particulate matter ≤2.5 µm (PM_2.5_), syndromic events, and hospitalizations. Sensitivity and positive predictive value were low for syndromic events, while specificity was high. Utilizing the larger sample of emergency departments, a strong day of week pattern and weak seasonal trend were observed for syndromic events and hospitalizations. These time-series were highly correlated after removing the day-of-week, holiday, and seasonal trends. The estimated percent excess risks in the cold season (October to March) were 1.9% (95% confidence interval (CI): 0.6, 3.2), 2.1% (95% CI: 0.9, 3.3), and 1.8% (95%CI: 0.5, 3.0) per same-day 10 µg/m^3^ increase in PM_2.5_ for cardiac-only syndromic data, cardiovascular syndromic data, and hospitalizations, respectively.

**Conclusions/Significance:**

Near real-time emergency department chief complaint data may be useful for timely surveillance of cardiovascular morbidity related to ambient air pollution and other environmental events.

## Introduction

The New York City (NYC) Department of Health and Mental Hygiene (DOHMH) implemented an active electronic prospective syndromic surveillance system in 2001 that monitors emergency department (ED) visits in near-real-time to identify temporal or spatial clusters in illness [1]. A main advantage of syndromic systems over traditional surveillance is timeliness. As in other jurisdictions where near-real-time tracking of emergency department visits is carried out, one motivation for their development was concern about biological terrorism. Most uses of these data involve tracking syndromes that may indicate communicable disease outbreaks, including the onset of the influenza season [2], . Syndromic surveillance has identified increases in gastrointestinal illness following a power outage [5], but applications of such near-real-time syndromic surveillance systems for tracking non-communicable health conditions that may be related to environmental events, such as heat related illness [6], [7], [8] have been more limited.

Cardiovascular disease (CVD) morbidity has been linked to ambient air pollution especially fine particles [9], [10], [11], [12], [13], influenza activity [14] public smoking bans [15], [16], and to natural and manmade disasters [17], [18], [19]. Typically these phenomena are investigated months or years after they occur as administrative hospital admission data become available. While syndromic surveillance of emergency department visits might allow more timely identification of these impacts, in some cases guiding public health response, the feasibility and utility of near-real-time CVD syndrome tracking using emergency department data has not been explored.

In this paper, we describe the methods of developing a CVD syndromic definition using data from emergency department visits in NYC and comparing temporal patterns in CVD events defined by syndromic coding to hospital discharges in NYC using discharge data collected by the Statewide Planning and Research Cooperative System (SPARCS) of New York State. We also examined the relationship between ambient fine particles and the CVD syndromic data and hospitalizations to test the comparability of environmental effects on these CVD morbidity indicators.

## Methods

### Ethics Statement

The New York City DOHMH Institutional Review Board approved this study involving existing data that could not be linked to individuals and were thus analyzed anonymously.

### ED Data Collection

Data from the EDs of 50 hospitals in New York City (comprising approximately 95% of all ED visits in New York City) is sent to the DOHMH daily via direct file transfer or as an email attachment. Electronic files contain date and time of each patient admission, age, sex, residential zip code, and the patient's reason of visit or chief complaint, recorded as a free-text field. The data do not contain personal identifiers such as name, birth date, or address. Table 1 summarizes the data sources used in this analysis.

**Table 1:** Description of data used in analyses.

Data Source	Number of institutions	Date range	Variables collected
Subset of Emergency Departments with ICD-9 Discharge Codes	11	1/1/2000–6/30/2002	Date of visit
			Hospital name
			Age
			Sex
			Chief complaint
			ICD-9 discharge code
All Emergency Departments	50	1/1/2004–12/21/2006	Date of visit
			Hospital name
			Age
			Sex
			Chief complaint
Hospitals	76	1/1/2004–12/21/2006	Date of admission
			Date of discharge
			Type of admission
			Hospital identification
			Age
			Sex
			ICD-9 discharge code

#### Syndrome coding ED data (11 hospitals, 2000–2002)

At the time this study was conducted, discharge diagnoses for emergency department visits were not routinely reported as part of the syndromic surveillance system. To compare chief complaint defined syndromes to discharge diagnoses, we used data from a subset (n = 11) of the 50 hospital EDs for the period January 1, 2000 through June 30, 2002, which included both chief complaint text and discharge diagnosis code, based on the International Classification of Diseases, Ninth Revision (ICD-9). Discharge diagnosis data before 2000 and after 2002 were unavailable. There were a total of 2,831,891 patient visits. We excluded 943,963 ED visits (33.3%) that had a missing or illegible chief complaint description, 3812 ED visits (0.3%) that were missing an ICD-9 diagnosis code, 3948 (0.1%) that were missing both, and 1,299,463 (45.9%) that were 39 years of age or younger given CVD outcomes are predominantly observed in older individuals. Thus our syndrome coding of ED data consisted of 580,841 (20.5%) patient visits with a valid chief complaint, ICD-9 diagnosis code, and recorded age of 40 years or older (median age: 54, interquartile range (IQR): 46–67).

#### Developing a Syndromic Definition

The New York City DOHMH has developed a syndromic surveillance system that uses ED visit chief complaint information and other non-identifying data elements reported electronically each day from hospitals in New York City. Chief complaint text is scanned to identify syndromes of interest classified as respiratory, sepsis, cold, diarrhea, rash, fever/influenza, asthma, and vomiting. Details of the methods used in this system have been published elsewhere [1]. We developed chief complaint text scanning criteria to identify possible CVD emergency department visits and compared trends in such visits to trends in hospital admissions from CVD causes.

We developed an algorithm that scans the free-text chief complaint field for character strings indicative of CVD events. CVD keywords include, for example, “heart attack,” “heart racing,” “chest pain,” “blood pressure,” “stroke,” etc. We included both events (e.g. myocardial infarction) and symptoms (e.g. chest pain) in our algorithm because the chief complaint text is completed by triage staff and, in some cases, records the patient's complaint verbatim while other descriptions correspond to a possible a diagnosis rather than a complaint. The coding algorithm is programmed to capture common misspellings and abbreviations of CVD keywords. The conditions included, comprised of a CVD keyword or combination of CVD keywords, were based on previous studies of air pollution/weather events and CVD outcomes [9], [11], and include ischemic heart disease, myocardial infarction, dysrhythmia, heart failure, hypertension, chest pain, stroke, and shortness of breath. Using our algorithms, binary variables were coded representing the presence or absence of one or more of each of the CVD condition keywords.

We then classified principal discharge ICD-9 codes in the range representing cardiovascular diseases (405–441) and symptoms (785–786) into seven categories corresponding to different keywords (Table 2). For example, the keyword “chest pain” corresponds to ICD-9 code *786.5* while “heart attack” corresponds to ICD-9 code 410.

**Table 2:** Agreement between CVD conditions and ICD-9 discharge codes, 2000–2002.

Conditions	ICD-9 Codes	Chief complaint keywords	Prevalence (%)	Sensitivity	Specificity	PPV
Myocardial infarction	410	Heart attack	0.2	0.007	0.999	0.010
Heart failure	428	CHF	<0.1	0.016	0.999	0.014
Dysrhythmia	427–429	Heartbeat	0.7	0.299	0.996	0.345
		Heart racing				
		Tachycardia				
Stroke	430–436	CVA	0.1	0.081	0.999	0.075
		Stroke				
Chest pain	411, 786.5	Angina	4.2	0.482	0.968	0.398
		Chest pain				
Shortness of breath	786.05	Short of breath	1.8	0.288	0.983	0.237
Hypertension	401–405	Blood pressure	0.6	0.119	0.997	0.193
		Hypertension				

+Prevalence defined by ICD-9 codes.

Our initial analysis proceeded in two phases. First, we evaluated the predictive value, sensitivity, and specificity of daily counts of individual CVD symptoms and conditions (i.e. ischemic heart disease/myocardial infarction, dysrhythmia, heart failure, hypertension, chest pain, stroke, and shortness of breath) using chief complaint and ICD-9 discharge code, the latter used as the gold standard. After evaluating individual symptoms and conditions, we estimated the predictive value, sensitivity, and specificity of seven individual or combinations of syndromic keywords (Table 2). After evaluating multiple combinations of the seven keyword categories, we defined two syndromes , one based on cardiac-only keywords (Syndrome 1) and the second based on a broader set of cardiovascular keywords (Syndrome 2), that had the best overall fit based on predictive value, sensitivity, and specificity (Table 3).

**Table 3:** Agreement between CVD syndromes and ICD-9 discharge codes, 2000–2002.

Syndrome	ICD-9 Codes	Chief complaint keywords	Prevalence (%)	Sensitivity	Specificity	PPV
Syndrome 1 (Cardiac)	410–429	Heart attack	4.6	0.380	0.970	0.379
	785–786	CHF				
		Heartbeat				
		Heart racing				
		Tachycardia				
		Angina				
		Chest pain				
Syndrome 2 (Cardiovascular)	405–441	Blood pressure	7.1	0.328	0.953	0.348
	785–786	Hypertension				
		Heart attack				
		CHF				
		Heartbeat				
		Heart racing				
		Tachycardia				
		Angina				
		CVA				
		Stroke				
		Chest pain				
		Short of breath				

#### Temporal analysis ED data (N = 50 hospitals, 2004–6)

We limited our primary analysis to 2004–2006 because fewer hospitals reported ED data prior to 2004 and because hospitalizations data after 2006 were unavailable to us during the analysis phase of this study. For the study period, there were a total of 9,689,368 ED visit records (before exclusions). We excluded 200,324 ED patient visits (2.1%) that were missing a chief complaint description, 6 (<0.1%) that were missing age, 5,645,386 (58.3%) with a recorded age of 39 years and younger, and 35,234 visits that were recorded between December 22, 2006 and December 31, 2006 because hospitalization data between these dates were incomplete. Thus, a total of 3,808,418 (39.3%) subject visits were included from ED visits data for further analysis (median age: 55, interquartile range (IQR): 47–69).

### Inpatient admission data

Hospital inpatient admission data were obtained from a statewide acute care hospital discharge database, known as SPARCS. This analysis used discharge records for New York City residents hospitalized at any New York State hospital during the calendar years 2004–6 (n = 3,774,537 discharge records before exclusions). An ICD-9 discharge code was available for all records. We excluded 1,512,485 (40.3%) patients 39 years of age and younger, 425,279 (11.3%) patients with a reported elective or unknown hospitalization type, 75,756 (2.0%) patients without an admission date, and 7400 (0.2%) hospitalizations between December 22 and December 31, 2006 because a large proportion of admissions near the end of that year may not have been discharged in 2006 and would not be included in our data. Thus, a total of 1,733,908 (45.9%) hospitalizations from SPARCS data sources between January 1, 2004 and December 21, 2006 are included in this analysis, with a median age of 66, (IQR: 53–78). The same method we used for classifying ED ICD-9 discharge codes was used for hospitalization discharge codes. To confirm our assumption that CVD outcomes occur primarily in older patients, we calculated the number of CVD-related hospitalizations by age group. Approximately 95% of individuals with a CVD-related hospitalization were> = 40 years of age.

### (1) Temporal analysis

We compared the patterns of temporal variation in daily counts of CVD syndromic classifications to counts of CVD-related non-elective hospital admissions using graphical displays (time-series plots and scatterplots) and time series models. In order to explore the day of week and seasonal trends, we plotted mean visits and standard errors by season and day of week. Furthermore, we created a holiday indicator given CVD-related ED visits and hospitalizations may be related to the work week. To compare the correlation between our CVD syndromic classifications and CVD-related hospitalizations, residuals from Poisson generalized linear models (GLMs), adjusting for seasonal/temporal trends, holidays, and day-of-week patterns, were estimated and plotted. The seasonal and temporal trends were estimated by fitting natural cubic splines of study days with 15 degrees of freedom (5 degrees of freedom per year); this “optimum” degrees of freedom was based on an evaluation of the sum of absolute values of partial auto-correlation function (up to 30 day lags) of the residuals [20], [21]. We calculated the percent deviance explained by season, day-of-week, and holiday patterns by estimating the deviance from a null GLM and a full GLM model with the season/temporal trend, day-of-week, or holiday term; the full deviance was subtracted from the null deviance and then divided by the null deviance. Finally, we fit time series models using a GLM separately for cold (October to March) and warm (April to September) weather months that included a seasonal smoothing function, day of week and holiday terms, and the count of CVD syndromic events with separate terms for lags 0–3 days as predictors of CVD hospitalizations. The percent increase in CVD hospitalizations and 95% confidence intervals (95% CI) were estimated per interquartile range increase in CVD syndromic counts. The goal was to assess the extent to which CVD syndromic events and hospital admissions co-vary temporally above and beyond any shared seasonal, day of week, and holiday patterns.

### (2) Analysis of Associations between Ambient Fine Particles and CVD Syndrome and Hospitalizations

In order to assess the usefulness of the CVD ED syndrome data as a health outcome indicator to study the impact of environmental risk factors, we investigated whether CVD ED syndrome counts and hospital admissions were temporally associated with concentrations of ambient fine particles with aerodynamic diameter less than or equal to 2.5 micrometer (PM_2.5_), which have been shown to exacerbate or trigger CVD events. PM_2.5_ data from all the monitors within a 20-mile radius of the geographic center of NYC were obtained from Environmental Protection Agency's Air Quality System. The daily average values from these monitors were used for analysis. We used a model specification similar to those used in recent air pollution epidemiological studies [11], [12]. A Poisson regression model was fitted to estimate percent excess risk of CVD ED syndrome counts per 10 µg/m^3^ increase in PM_2.5_, adjusting for: (1) temporal trends and seasonal cycles using natural cubic splines (NS) of study days using 7 degrees of freedom per year; (2) immediate temperature effects using NS of same-day temperature with 3 degrees of freedom; (3) delayed temperature effects using NS of the average of lag 1- through 3-day temperature with 3 degrees of freedom; (4) day-of-week, and accommodating over-dispersion. We examined PM_2.5_ effects at lag 0 through 3 days. Because PM_2.5_ effects may be modified by season, we also analyzed the data for warm (April–September) and cold (October–March) seasons. All analyses were conducted using SAS (version 9.1, SAS Institute Inc., Cary, NC) and R (version 2.9.0, R Development Core Team).

## Results

### Developing a syndromic CVD definition

Of 580,841 ED visits in our syndrome coding data, a total of 44,427 (7.6%) were classified into 1 of the 7 individual CVD conditions or symptoms defined by ICD-9 diagnosis code (Table 2). The most common CVD-related event was chest pain (n = 24,523) and the least common was heart failure (n = 165). Sensitivity varied widely, from 0.007 for MI to 0.482 for chest pain. Similarly, positive predictive value (PPV) varied greatly, from 0.010 for MI to 0.398 for chest pain. Specificity was high for all individual syndromic criteria. For our two broader CVD syndromic definitions, we observed low sensitivity and PPV (<0.40) when compared to the corresponding ED ICD-9 discharge code, though specificity for both definitions was high (>0.95) (Table 3).

### CVD Syndromic Data from 50 NYC Emergency Departments and 76 NYC Hospitals, 2004–2006

The mean number of citywide ED visits per day among adults age 40 and above between January 1, 2004 and December 21, 2006 was 3507 (range 2061 to 4585). During this same period, a total of 337,823 ED visits met syndromic definition #1, an indicator of cardiac condition, (mean: 311 visits per day, range: 163–525, approximately 9% of all ED visits); while 501,155 ED visits met syndromic definition #2, corresponding to a broader range of cardiovascular conditions, (mean: 461 visits per day, range: 260 to 727, approximately 13% of all ED visits). A daily mean of 1597 hospital visits (range: 858 to 2080) were recorded between 2004 and 2006. Of these hospitalizations, 393,063 were observed to be CVD-related (mean: 362 hospitalizations per day, range: 195 to 511, approximately 23% of all hospitalizations).

**Figure 1:**
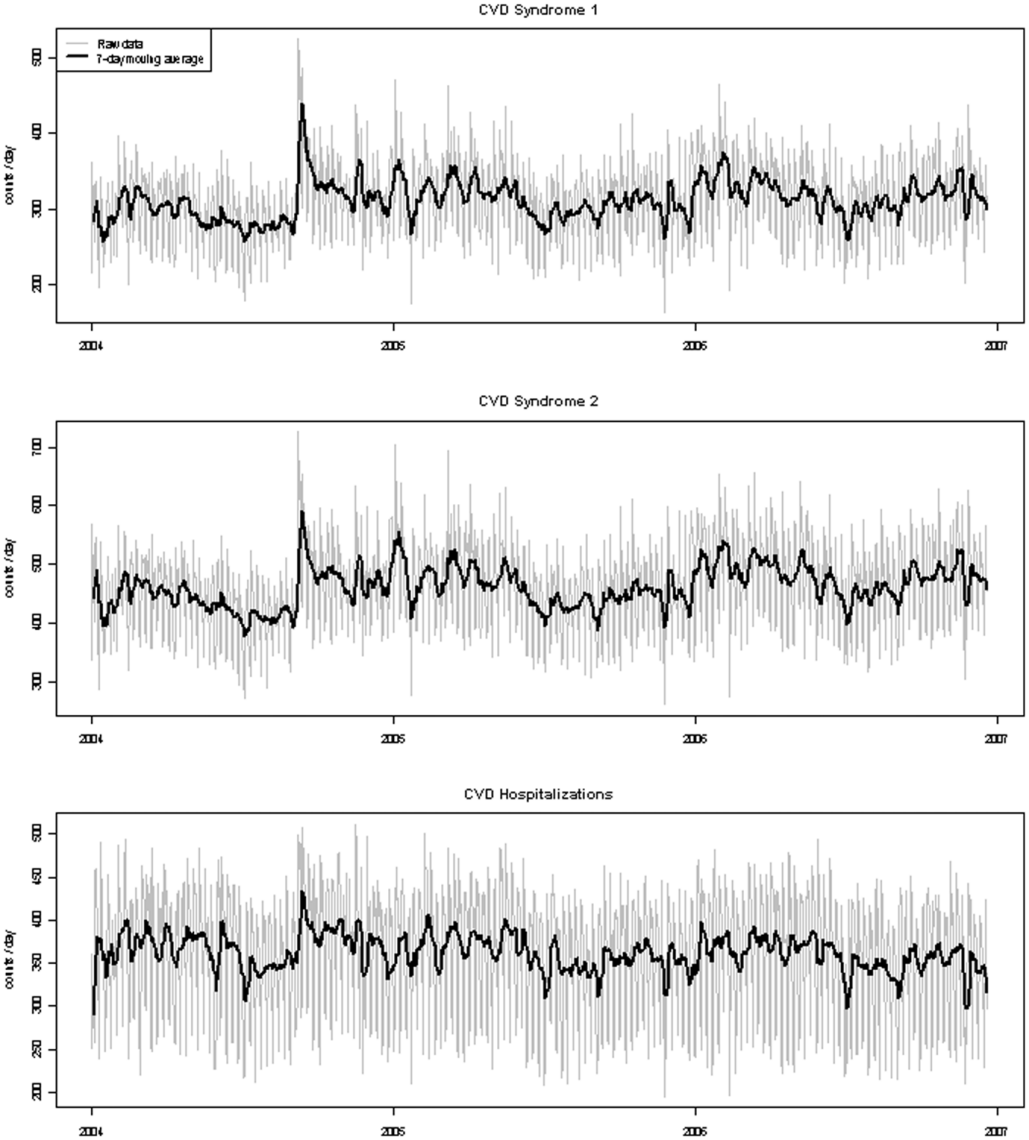
Daily counts and 7-day moving average of cardiovascular syndrome 1, syndrome 2, and cardiovascular-related hospitalizations, 2004–2006.

Daily counts and 7-day moving average of Syndrome 1, Syndrome 2, and CVD-related hospitalizations for 2004 to 2006 is shown in Figure 1. A weak seasonal pattern is observed as CVD syndromic ED visits rise modestly in the colder months and decline in the warmer months. The difference in mean CVD visits between summer and winter seasons is less than 10% for both syndromic definitions (298 in summer/320 in winter for Syndrome 1 and 438/477 for Syndrome 2).

**Figure 2:**
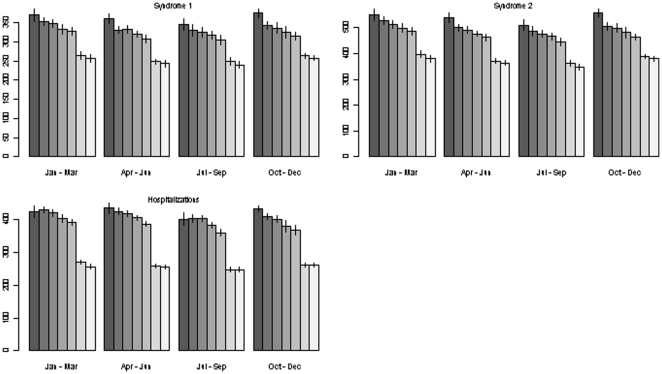
Mean counts of cardiovascular syndromes 1 and 2, and cardiovascular-related hospitalizations, by day of week (Monday through Sunday) and season, 2004–2006.

A strong day of week pattern was observed for CVD syndromes and hospitalizations. Figure 2 shows mean and standard error of the CVD ED syndrome and hospitalizations by day of week and season. CVD visits to the ED were highest on Monday (range: 8–18% higher than the weekly average) and gradually decline before experiencing their nadir on the weekend (range: 17–21% below the weekly average). Hospitalizations follow a similar pattern, as Monday admissions were 15–21% higher than the weekly average and weekend admissions were 27–31% below the weekly average.

The amount of deviance explained by seasonal, day of week, and holiday trends differs greatly (Table 4). Seasonal and holiday patterns were found to explain little of the variance seen in the time series while day of week patterns explained approximately 57% and 62% of deviance in the time series of Syndromes 1 and 2, respectively, and 80% in the time series of hospitalizations. Holidays tended to coincide with the lowest syndromic and hospitalization counts shown in Figure 1, though the small amount of variance explained by holidays was expected given only ten holidays occur per year.

**Table 4:** Deviance explained by temporal trends for syndrome 1, syndrome 2, and hospitalizations, 2004–2006.

Condition	Deviance explained (%)
Syndrome 1	
Day of week	56.5
Season	10.0
Holiday	1.7
Syndrome 2	
Day of week	61.5
Season	10.0
Holiday	1.5
Hospitalizations	
Day of week	80.0
Season	2.8
Holiday	2.3
Syndrome 1	67.3
Syndrome 2	70.9

**Figure 3:**
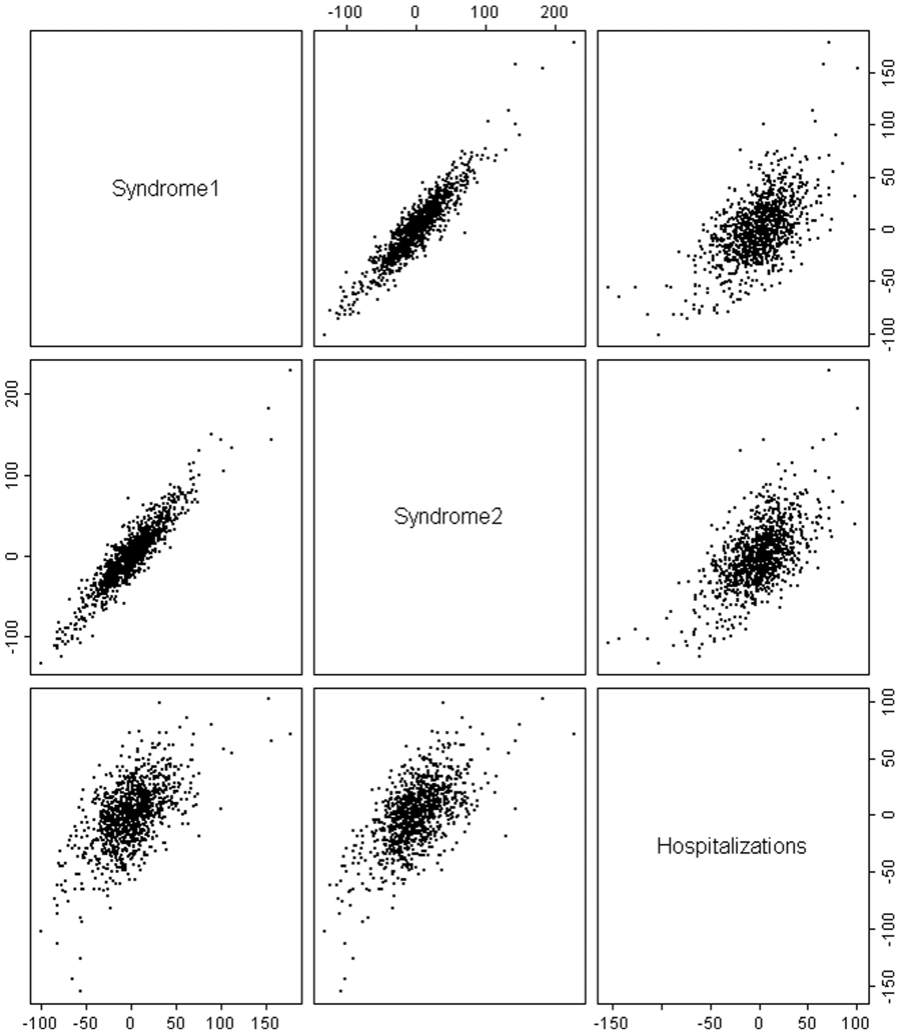
Scatter plot of the residuals (from seasonal, day-of-week, and holiday trend fit) between syndrome 1, syndrome 2, and cardiovascular-related hospitalizations.

To explore the temporal correlation between the CVD syndromic definitions and hospitalizations by season, we plotted lagged cross-correlations of the residuals from our GLMs. We observed a strong same-day cross-correlation between the syndromic definitions and hospitalizations that appeared to be consistent throughout all seasons (results not shown). We also examined the correlation between our syndromic definitions and CVD-related hospitalization after adjusting for the strong day of week pattern and weaker seasonal and holiday variation. Scatter plots of the residuals shows moderate correlation between the two syndromes and hospitalizations (Figure 3). Daily counts of ED visits for syndromes 1 and 2 were observed to have a correlation of 0.59 and 0.61, respectively, with CVD-related hospitalization counts. Finally, the daily variation in CVD-related hospitalization was observed to be associated with ED visits for the CVD syndromic definitions during the warm and cold seasons. For Syndrome 1, an interquartile range increase in CVD syndrome counts was associated with a 10% increase in CVD-related hospital admissions during the warm season (95% CI: 9–12%) and a 14% increase in CVD-related hospitalization admissions in the cold season (95% CI: 13–16%, Table 5). Risk of hospital admission was similar for Syndrome 2. Higher CVD syndrome counts for lag 1–3 were also predictive of higher CVD hospitalization rates, though temporal autocorrelation in each time series was likely responsible for part of this observed association.

**Table 5:** Percent increase and 95% confidence intervals of CVD-related hospital admissions per interquartile range increase in CVD syndromic counts, 2004–2006.

	Syndrome 1	Syndrome 2
Season	% increase* (95% CI)	% increase* (95% CI)
Cold	14.1 (12.5–15.7)†	16.0 (14.2–17.8) †
Warm	10.0 (8.8–11.5) †	12.5 (10.8–14.1) †

*Adjusted for seasonal (5df/yr), day-of-week, and holiday trends.

† p<0.05.

**Figure 4:**
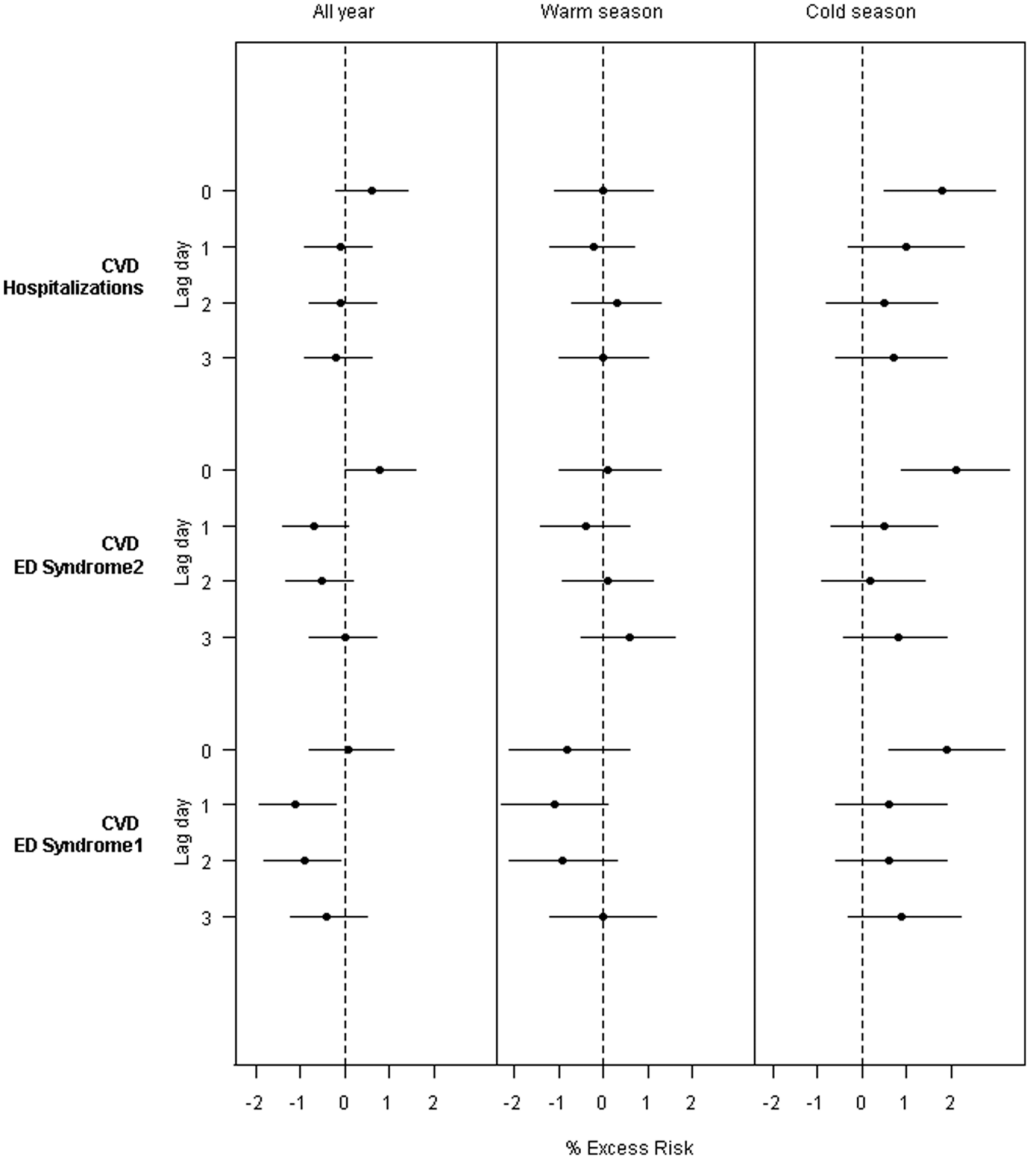
Percent excess risk per 10 µg/m^3^ increase in PM_2.5_ at lag 0 through 3 days for warm (April–September) and cold (October–March) seasons, adjusted for temperature, seasonal trends, and day-of-week.

In the analysis of associations between PM_2.5_ and the CVD health outcomes, we found that both lag structure and seasonal patterns of associations between PM_2.5_ and these outcomes were very similar: the strongest associations occurred on lag 0 day and in colder season (Figure 4). The estimated percent excess risks at lag 0 day in cold season were 1.9% (95% CI: 0.6, 3.2), 2.1% (95% CI: 0.9, 3.3), and 1.8% (95%CI: 0.5, 3.0) per 10 µg/m^3^ increase in PM_2.5_ for Syndrome 1, Syndrome 2, and hospitalizations, respectively.

## Discussion

We found that emergency department chief complaint data has only fair predictive value for CVD diagnoses. However, despite substantial misclassification in individual cases, temporal patterns in CVD syndrome ED visits are quite consistent with those observed for CVD hospital admissions. As discussed below, we feel these findings show that CVD syndrome tracking using emergency department data, while having important limitations, may be useful for near-real time tracking CVD morbidity in relation to environmental events – providing more timely indication of departures from expectation than are available from traditional diagnosis based surveillance using hospital administrative data.

Chief complaint descriptions are brief (generally less than 50 characters long) and are typically recorded before a patient has been evaluated and treated, thus not capturing all information relevant a diagnosis. The poor overall agreement between our syndromic definitions and corresponding discharge diagnosis, as defined by the ED, is not surprising, given conditions like heart failure and dysrhythmia are not commonly found in chief complaints while other complaints, namely chest pain and shortness of breath, may be CVD-related but are non-specific enough to be related to many non-CVD events. For example, a common chief complaint among ED patients with a CVD-related ICD-9 discharge code was “I am not feeling well.” Our finding is consistent with other studies comparing chief complaint and ICD-9 discharge code in other syndromic studies of non-CVD events [3], [22].

Temporal trends of our CVD syndrome ED visits, and CVD-related hospitalizations to a lesser extent, show a somewhat weak seasonal variation. This finding is generally consistent with previous studies documenting seasonal variation in CVD morbidity [23], [24], [25], [26], though the seasonal trend we observed was not the dominant feature of our time-series. In general, CVD events, particularly myocardial infarction, peak in the colder months and reach their nadir in the warmer months, and increased risk for CVD events may be higher in warm climates that experience cold temperatures [27]. While prior studies primarily focused on hospital admissions and not ED visits, it is encouraging that the seasonal pattern of our two CVD syndromic definitions reflects the general pattern observed among CVD-related hospitalizations. An anomalous increase of ED and hospital CVD events in early September 2004 may be explained by capture of CVD symptoms and concerns following President Bill Clinton's coronary artery bypass surgery in New York on September 6, 2004 and does not appear to reflect a weather event or other type of event. The observed increase of CVD syndromic events and CVD-related hospital admissions further suggests our syndromic definition is able to effectively capture and describe sudden temporal CVD anomalies.

We found variance among CVD events is driven primarily by day of week patterns, particularly by the start of the work week. Similarly, several [28], [29], [30], but not all [25], studies show that CVD events peak on Monday and gradually decline to a minimum of events on the weekend, suggesting a consistent day of week pattern for CVD events. It is generally thought that the start of the work week leads to increased stress levels which in turn increases risk of a cardiac event [31], [32], [33]. This interpretation is supported by the large decline in syndromic ED visits and CVD-related hospitalizations we observed when Monday is a holiday. It is also possible that the increase in visits on Monday could be related to a delay in seeking care during the weekend and holidays. Some studies have observed a larger seasonal variation in CVD events compared to weekly variation [25], [29], while others have found no day of week pattern [28], although only acute myocardial infarction hospital admissions were typically analyzed. The inclusion of other CVD events, along with myocardial infarction, into our syndromic and hospitalization analyses may be responsible for this difference in day of week patterns we observed with these latter studies. The daily (and seasonal) time-series agreement between our syndromic definitions and hospital discharges may suggest those ED-based CVD events that we capture are more serious, thus reflecting temporal patterns seen in CVD-related hospitalizations. Conversely, it is also possible that those with less severe CVD symptoms may not seek ED care.

The two syndromic definitions were modestly correlated with hospitalizations, even after accounting for seasonal, day of week, and holiday patterns. We stratified our analyses by warm/cold season given the association of CVD events with temperature. Both syndromic definitions were found to predict risk of CVD-related hospitalization during the warm and cold season. This finding was strongest on the same day which was expected given our observation of a strong same-day cross-correlation of our CVD syndromes with CVD-related hospitalizations. Overall this suggests that the CVD syndromic definitions we developed are good indicators of short-term temporal variation in CVD-related hospitalization, beyond that expected due to seasonal, day of week, and holiday variation.

We found that the CVD syndromic definitions and CVD hospitalizations showed a very similar pattern of associations with PM_2.5_. These associations were stronger in the colder season, which is consistent with a recent multi-city study reporting that associations between PM_2.5_ and CVD hospitalizations were stronger in Northeast and in the cold season [34]. The magnitude of PM_2.5_ effects estimated was also comparable between the syndromic definitions and hospitalizations. Further, our results agree with a prior study from Australia, which showed an effect of PM_2.5_ on risk of CVD syndromic visits to the ED [8]. The result supports the use of CVD ED syndromic series as a near-real time health outcome indicator of CVD morbidity in response to exposure to environmental risk factors.

There are several strengths to this study. We achieved near-complete capture (approximately 95%) of emergency department chief complaint data in New York City between 2004 and 2006. In addition, specificity of our syndromic definitions was high. We also utilized ICD-9 discharge codes rather than admission codes for both ED visits (when available) and hospital visits. There is greater potential for a miscoded diagnosis during admission versus discharge, due to either nonspecific symptoms and/or unconfirmed diagnoses, and we were able to minimize this potential misclassification by utilizing discharge codes. This study also has several limitations. We excluded approximately two-thirds of ED visits due to age restrictions and missing or illegible chief complaint data. However, this did not obscure the temporal patterns that paralleled those in CVD hospital admissions. Second, we assumed ICD-9 discharge codes from ED and hospital records represented correct diagnoses. While ICD-9 discharge codes have been found to correspond fairly well to true disease [2], [4], correlation is not perfect. Indeed, assignment of ICD-9 codes may be more related to billing practices than true disease [35]. However, it is unlikely that such misclassification of discharge diagnoses could explain the temporal tracking of CVD syndromic events with CVD hospital discharge ICD-9 discharge codes.

In summary, near real-time monitoring of emergency department chief complaint data may be useful for timely surveillance of CVD morbidity. A primary purpose of syndromic surveillance is to detect citywide increases in illness [36], and one of the primary advantages of chief complaint data over ICD-9 discharge data is timeliness. Given that chief complaint information is generally available to the NYC DOHMH within 24 hours after a visit to the ED, we have the ability to recognize sudden changes in CVD-related events. While an ICD-9 diagnosis code may have a higher agreement with one's true condition compared to a chief complaint that is recorded before physician examination, the lag time involved in obtaining hospital administrative data currently precludes its use in near real-time surveillance. While further evaluation is needed, near-real-time CVD surveillance could provide valuable situational awareness in the acute phase or immediate aftermath of natural or manmade disasters involving exposures that could exacerbate cardiovascular disease.
